# Mentalising Moderates the Link between Autism Traits and Current Gender Dysphoric Features in Primarily Non-autistic, Cisgender Individuals

**DOI:** 10.1007/s10803-020-04478-4

**Published:** 2020-04-01

**Authors:** Aimilia Kallitsounaki, David Williams

**Affiliations:** School of Psychology, Keynes College, University of Kent, Canterbury, CT2 7NP UK

**Keywords:** Autism, Gender dysphoria, Gender identity, Mentalising, ToM

## Abstract

The co-occurrence between autism and gender dysphoria has received much attention recently. We found that, among 101 adults from the general population number of autism traits, as measured using the autism-spectrum quotient was associated significantly with recalled and current gender dysphoric traits. Furthermore, performance on an objective measure of mentalising, such as the “Reading the Mind in the Eyes” test was associated with current gender dysphoric traits, but most importantly it moderated the relation between number of autism traits and number of current gender dysphoric traits, such that the association was significant only when mentalising ability was relatively low. Results suggest mentalising may represent a contributing factor to the relation between autism and gender dysphoric traits in the general population.

**Electronic supplementary material:**

The online version of this article (10.1007/s10803-020-04478-4) contains supplementary material, which is available to authorized users.

Autism spectrum disorder (henceforth autism) is a neurodevelopmental disorder, which is defined in terms of severe behavioural difficulties with social-communication and restricted, repetitive pattern of interests and behaviour (American Psychiatric Association 2013). Autistic people frequently have co-occurring disorders of one form or another (e.g., Simonoff et al. 2008), but one particular “comorbidity” has received much attention recently. Gender dysphoria, formally known as gender identity disorder in DSM-IV-TR (American Psychiatric Association 2000), is a psychiatric condition characterised by an incongruence between one’s own assigned gender at birth and one’s experienced/reported gender, which is accompanied by negative emotions about the characteristics of one’s assigned gender (American Psychiatric Association 2013). Despite clear phenotypic differences between autism and gender dysphoria, recent research findings suggest the existence of a link between them (e.g., Strang et al. 2018; van der Miesen et al. 2018a).

Within the autism population, retrospective chart reviews have shown that affected people are significantly more likely than neurotypical individuals to express the wish to be the opposite gender (Janssen et al. 2016; May et al. 2017; Strang et al. 2014; van der Miesen et al. 2018c). Furthermore, in a recently published study, George and Stokes (2018) used the Gender Identity/Gender Dysphoria Questionnaire (Deogracias et al. 2007) and found that adults with autism reported significantly increased gender dysphoric traits relative to neurotypical individuals.

Within the gender dysphoria population, research has shown that the rate of a diagnosis of autism is significantly higher than the population estimate of autism (de Vries et al. 2010; Heylens et al. 2018), which is approximately one percent (Baird et al. 2006). Even when people with gender dysphoria do not have clinical diagnoses of autism, many still show an increased number of autism traits compared to neurotypical individuals (Akgül et al. 2018; Jones et al. 2012; van der Miesen et al. 2018b; but see Nobili et al. 2018; Turban 2018 and Turban and van Schalkwyk 2018).

The above studies involved diagnosed cases of autism and gender dysphoria, respectively, using a variety of research designs. Yet, both autism and gender dysphoria are considered dimensional conditions (e.g., Constantino and Todd 2003; Ehrensaft 2018; Ronald et al. 2006). As such, adopting an individual differences approach among individuals from the general population can tell us something important about the nature of autism and gender dysphoria themselves (e.g., Nicholson et al. 2018; Williams et al. 2018a, b, c).

To date, only one study has examined the overlap between autism and gender dysphoria in adults from the general population. George and Stokes (2018) found a positive and significant association between the number of self-reported gender dysphoric traits and the number of autism traits, as measured with the Autism-spectrum Quotient (AQ; Baron-Cohen et al. 2001b) among neurotypical individuals with an AQ score below 32. One aim of the current study was to attempt to replicate George and Stokes’ (2018) finding of a significant association between number of autism traits and number of gender dysphoric traits among adults from the general population. Given concerns about the reliability of results in psychological research, independent replication serves a crucial function in the discipline (e.g., Amir and Sharon 1990; Makel et al. 2012; Pashler and Wagenmakers 2012). The current study also moved beyond George and Stokes’ by including a second behavioural measure that assessed *retrospective* childhood crossgender identity, rather than only *current* gender dysphoric traits. Such a historical approach would increase our confidence that gender dysphoric traits measured in the current study reflect persistent gender identity difficulties that started early in life. Perhaps most important, the current study moves beyond previous studies in this domain by including an objective cognitive measure of mentalising.

What relatively little research has been conducted on this topic appears to provide evidence of a link between autism and gender dysphoria, but there is an ongoing debate on whether that link is anything more than superficial (Strang et al. 2018; Turban 2018; Turban and van Schalkwyk 2018; van der Miesen et al. 2018a). Indeed, behavioural overlap between two disorders represents only a weak source of evidence that the two disorders are truly comorbid, because any given behaviour (including self-reported behavioural traits) can have a number of different underlying causes (see Williams 2017; Williams et al. 2008; Williams and Lind 2013).

Arguably, it is only if the overlapping behavioural features have the same underlying cause that we should consider the two conditions comorbid and, as Williams (2017, p. 274) notes, “just because behaviourally-defined disorders A and B co-occur in a person does not mean that the underlying causes of those disorders are the same as the causes of A or B in isolation”. For example, it may be that when the behavioural features of gender dysphoria are apparent in a person with a primary diagnosis of autism, those features have a different underlying cause (cognitive/neurobiological/genetic/environmental) to the underlying cause of behavioural features of gender dysphoria in a person with a diagnosis of gender dysphoria. It may be, for example, that the autism itself predisposes affected individuals to develop gender dysphoric features (or vice versa). In that case, gender dysphoria in people with autism would represent a “phenomimic” of gender dysphoria (see Bishop 2006, 2010). In other words, gender dysphoria might be a different entity that just appears at the behavioural level to be the same in both autism and gender dysphoria, because it is defined by behaviour only. Behaviour is a sub-optimal means of diagnosing disorders and so we may be dealing with two different psychopathological entities that just appear to be similar.

At the psychological level, one candidate mechanism proposed as an explanation for the apparent overlap between autism and gender dysphoria is diminished *mentalising* (Glidden et al. 2016; Jacobs et al. 2014; Van Der Miesen et al. 2016, 2018c). Mentalising (or else Theory of Mind; ToM) is the ability to represent mental states in order to explain and predict behaviour (Premack and Woodruff 1978). It is widely considered to be diminished in autistic people and to contribute to the social-communication impairments that are diagnostic of the disorder (see Brunsdon and Happé 2014). Moreover, ToM difficulties may qualify as a cognitive marker of autism, given that they correlate negatively with autism severity (Brunsdon and Happé 2014), run in families of people with autism (Gliga et al. 2014), and are apparent even when behavioural features have apparently resolved (Kelley et al. 2006). In a recently published study, Stagg and Vincent (2019) examined mentalising in transgender and nonbinary people. They found that neurotypical individuals had significantly better ToM skills compared to nonbinary people, but equivalent to transgender individuals.

Theoretically, diminished ToM could contribute to gender incongruence/dysphoria in a number of ways. First, it could influence gender constancy, which is the representation of gender as a construct that can remain stable even when temporary changes in physical appearance take place (e.g., a boy does not become a girl as soon as he grows long hair or puts on a dress). Gender constancy is central to theories of gender identity development (e.g., Kohlberg 1966) and bears close resemblance to the ability to distinguish between appearance and reality, which is a core aspect to ToM. Indeed, among typically developing children, ToM ability is associated positively with performance on tests of gender constancy (Trautner et al. 2003; Zmyj and Bischof-Köhler 2015), and negatively with use of gender stereotypes (Rizzo and Killen 2018). Second, any internalisation of gender-related attributes requires an accurate awareness of those attributes in others. It seems theoretically plausible to suggest that the development of gender identity would be affected by a difficulty representing gender-related internal states/traits in others. In such a circumstance, a child would likely pay less attention to potentially misleading physical cues (e.g., height, hair length etc.) when forming gender schemas. Third, it is well known that diminished ToM leads to reduced experience of self-conscious emotions (e.g., embarrassment/shame) (Hobson et al. 2006). Self-conscious emotions contribute to a desire for social conformity (we conform to society’s expectations in part to avoid the feelings of embarrassment/shame that results from others judgments when we do not conform). As such, these emotions may lead to a desistence of gender incongruent behaviours in children who have a tendency toward an incongruent identity.

In the current study, we used an individual differences approach to investigate whether mentalising plays a role in the suggested link between autism and gender dysphoria. Participants completed online a self-report measure of autism traits (the AQ; Baron-Cohen et al. 2001b) and a widely used cognitive-experimental task of mentalising (the Reading the Mind in the Eyes; RMIE; Baron-Cohen et al. 2001a). In addition, they completed a self-report measure of retrospective childhood crossgender identity (the Recalled Childhood Gender Identity/Gender Role Questionnaire; RCGI; Zucker et al. 2006) and a self-report measure of gender dysphoric traits (the Gender Identity/Gender Dysphoria Questionnaire; GIDYQ; Deogracias et al. 2007).

First, we aimed to examine the extent to which the number of self-reported autism traits is associated with *recalled* childhood crossgender identity/behaviour and/or *current* gender dysphoric traits. To our knowledge, no study has examined the link between autism and retrospective childhood crossgender identity. We predicted that AQ score would be negatively associated with both RCGI and GIDYQ score (lower scores on RCGI and GIDYQ are indicative of more recalled childhood crossgender identity/behaviour and more current gender dysphoric traits). Second, given the theoretical link between mentalising and the process of gender identity formation we predicted that RMIE score would be positively associated with both RCGI and GIDYQ score. Third, we tested the hypothesis that mentalising modulates the predicted association between autism traits and gender dysphoric traits by conducting a moderation analysis. Moderation analysis examines whether the direction or strength of the relation between two variables is influenced by a third variable. In the current study, we predicted that performance on the mentalising measure would moderate the relation between autism traits and current gender dysphoric traits.

## Method

### Participants and Procedure

One hundred and one adults (50 female) participated in the current study through the Amazon’s online crowdsourcing platform MTurk. The average age of the sample was 36.93 (*SD* = 10.11; range 22 to 70) years. No participant reported incongruency between their assigned gender at birth and their experienced gender. Ninety-four percent of participants reported being native English speakers. Thirteen participants had a formal diagnosis of autism, according to self-report. All participants took part after they had given written, informed consent and received compensation for their time. This study was approved by Kent School Research Ethics Committee.

### Measures

#### Autism-Spectrum Quotient

The AQ (Baron-Cohen et al. 2001b) is a reliable self-report questionnaire that measures autism traits in clinical and non-clinical populations (e.g., Ruzich et al. 2015; Williams et al. 2018b). Participants are presented with 50 self-referential statements (e.g., “I find social situations easy”) and they are asked to indicate their agreement with each statement, using a four point Likert scale that ranges from “definitely agree” to “definitely disagree”. The AQ sum score ranges from zero to 50 and a value of 26 or above indicates a clinically significant number of autism traits (Woodbury-Smith et al. 2005). Its test–retest reliability scores range from.70 to.95 (e.g., Baron-Cohen et al. 2001b; Broadbent et al. 2013) and it has shown convergent validity with the Social Responsiveness Scale in clinical (*r* = .64; Armstrong and Iarocci 2013) and non-clinical samples (*r* = .55; Ingersoll et al. 2011).

#### Reading the Mind in the Eyes

The RMIE (Baron‐Cohen et al. 2001a) is a widely used measure of mentalising. Participants are presented with a series of 36 photographs each showing the eye region of people who are experiencing a particular emotion/thought. Participants are asked to choose which one of four presented words best describes the emotional/mental state of the depicted person. Scores range from zero to 36, with higher scores indicating better task performance. The task has been employed in over 250 studies, and shows acceptable test–retest reliability of over.60 in most studies (e.g., Dehning et al. 2012; Fernández-Abascal et al. 2013; Voracek and Dressler 2006), including over long periods of time (e.g., Fernández-Abascal et al. 2013 report an intraclass coefficient of.63 for 1 year test–retest reliability). Task performance clearly distinguishes groups of participants with and without autism, even when participant groups are closely matched for verbal ability (e.g., Nicholson et al. 2019; Williams et al. 2018b) and is associated negatively with the number of autism traits shown by individuals in large population studies (e.g., Baron-Cohen et al. 2001b). Performance on the task is also correlated with performance on other measures of ToM, even after the influence of IQ is controlled statistically (e.g., Jones et al. 2018), and is associated with activation of a well-established ToM network of brain regions including the temporo-parietal junction and medial pre-frontal cortex (see Schurz et al. 2014).

#### The Recalled Childhood Gender Identity/Gender Role Questionnaire

The RCGI (Zucker et al. 2006) is a 23-item self-report measure that provides a retrospective assessment of childhood crossgender behaviour and closeness to parents in the first 12 years of life (e.g. “As a child, my favorite playmates were: a. always boys, b. usually boys, c. boys and girls equally, d. usually girls, e. always girls, f. I did not play with other children”). For the purposes of the current study, participants were asked to respond only to the 18 items comprised factor one in the original Zucker et al.’s (2006) study, using a five point scale. These items particularly assess gender role behaviour and gender identity. The questionnaire has parallel male and female versions and as such, participants completed the version according to their assigned gender at birth. A mean score was calculated for each participant, with means scores ranging from one to five and with lower scores denoting more childhood crossgender identity and behaviour. With respect to its psychometric properties, RCGI has shown convergent validity with the GIDYQ, *r* = .70 (Singh et al. 2010).

#### Gender Identity/Gender Dysphoria Questionnaire for Adolescents and Adults

The GIDYQ (Deogracias et al. 2007) is a 27-item self-report measure of gender identity and gender dysphoria. The GIDYQ presents participants with a series of questions about their feelings, wishes, thoughts and behaviours regarding their assigned gender at birth and their experienced gender within a one year period (e.g., “In the past 12 months, have you felt uncertain about your gender, that is, feeling somewhere in between a woman and a man?”). The questionnaire has two analogous versions, one for males and one for females. In the current study, participants completed the version that was consistent with their assigned gender at birth and responded to each of the items using a five point scale. A mean score was calculated for each participant, with scores ranging from one to five and with lower scores indicating increased gender dysphoric traits. A mean score of three is the cut-off point that indicates clinically significant levels of gender dysphoria (Deogracias et al. 2007). The original validation study showed that this self-report measure is reliable (Cronbach's alpha = .97) and that the sensitivity of the cut-off point is 90.4% among people with a diagnosis of gender dysphoria and its specificity 99.7% among a university students sample (Deogracias et al. 2007).

### Statistical Analysis

According to a well-documented dimensional approach (namely “broad autism phenotype”), autism is a spectrum disorder that ranges from people in the general population with low levels of autism traits to people who hold a clinical diagnosis of autism (e.g., Bolton et al. 1994; Goldberg et al. 2005; Le Couteur et al. 1996; Murphy et al. 2000; Pickles et al. 2000; Piven et al. 1997; Szatmari et al. 2000). Therefore, in the current study all participants were included in the analyses described below, independently on whether they reported possession of a formal diagnosis or not (please see the Supplementary Material for the results of the main analyses of the current study, when autistic participants were excluded). An alpha level of .05 was used to determine statistical significance.

To examine the relations among autism traits, recalled childhood crossgender identity/behaviour, current gender dysphoric traits, and mentalising, a series of zero-order correlations was conducted involving scores on the AQ, RCGI score, the GIDYQ, and RMIE. Following the suggestion of an anonymous reviewer, a series of post-hoc correlation analyses was conducted among AQ subscales, GIDYQ, and RCGI (for the results please see the Supplementary Material). Coefficients *r* are reported as measures of effect size (≥ .10 = small effect, ≥ .30 = moderate effect, ≥ .50 = large effect; Cohen 1992). Furthermore, a moderation analysis was employed to test the hypothesis that RMIE moderates the predicted relation between autism traits and current gender dysphoric traits. To perform this analysis we used PROCESS v3.3 operated in SPSS, employing bootstrapping with 5,000 resamples (Hayes 2018). To mitigate the potential risk of multicollinearity, products of interactions were mean centred prior to analysis (Dawson 2014).

In moderation analysis, it is crucial to investigate whether there is a significant interaction between a focal variable (F) and a moderator variable (M), when predicting an outcome (Y) variable (Hayes and Matthes 2009). In our moderation analysis, we examined whether there was a significant interaction between AQ score (F) and performance on the RMIE (M), when predicting current gender dysphoric traits (Y). If so, this result would imply that the predicted association is present to a lesser degree (or not at all) in participants with a high RMIE score than people with a low RMIE score (or vice versa).

According to standard practice, in order to understand the conditions, or else the values of the moderator under which the relation between a focal variable and an outcome variable holds its statistical significance or its effect size, the significant interaction should be further probed (Hayes and Matthes 2009). The “gold standard” method to probe the source of a significant interaction effect in a moderation analysis, *without* losing power by dichotomising continuous predictors is to perform simple slopes analysis (Field 2013; Hayes and Matthes 2009). To do so, we employed the widely-used “pick-a-point” approach (e.g., Bauer and Curran 2005; Dawson and Richter 2006), which involves probing interactions at one standard deviation above (+1SD) and below (-1SD) the mean for the moderator (Aiken et al. 1991).

In a series of exploratory analyses, we also compared the data from the 13 participants who reported possession of a formal diagnosis of autism to the data from the remaining 88 non-autistic participants. To examine between-group (autistic/non-autistic) differences in the number of self-reported autism traits, recalled gender dysphoric traits, current gender dysphoric traits, and in performance on the RMIE, independent samples *t*-tests were conducted. In cases when the homogeneity of variance assumption was violated results from the Welch’s *t*-test were reported (Field 2013). Cohen’s *d* values were reported as measures of effect size (≥ .20 = small effect, ≥ .50 = moderate effect, ≥ .80 = large effect; Cohen 1969). Finally, a Fisher's exact test was also employed to explore the extent to which there was a significant association between the diagnostic group (autistic/non-autistic) and GIDYQ score (above clinical threshold/below clinical threshold). A phi coefficient was reported as measure of effect size. Its values fall between zero and one and is interpreted as coefficient *r*.

## Results

Table 1 presents the results from the correlation analyses conducted to examine the relations among autism traits, recalled childhood crossgender identity/behaviour, current gender dysphoric traits, and mentalising.

**Table 1:** Bivariate correlations among AQ, GIDYQ, RCGI, and RMIE

Variables	1	2	3	4
1. AQ	–			
2. GIDYQ	− .32**	–		
3. RCGI	− .33**	.53***	–	
4. RMIE	− .18*	.70***	.33**	–

*N* = 101

*AQ* autism-spectrum quotient, *GIDYQ* Gender Identity/Gender Dysphoria Questionnaire (low scores = more gender dysphoric traits), *RCGI* the Recalled Childhood Gender Identity/Gender Role Questionnaire (low scores = more childhood crossgender identity), *RMIE* reading the mind in the eyes task

*p < .05 (one-tailed), **p < .01, ***p < .001

As predicted, AQ score was negatively and significantly correlated with both RCGI and GIDYQ mean score (i.e., the more autism traits, the more recalled and current gender dysphoric traits). Furthermore, RMIE score was positively and significantly correlated with RCGI score (i.e., the poorer the RMIE performance, the more the recalled gender dysphoric traits), but this was the only association that lost its significance when participants who reported a formal diagnosis of autism were excluded from the analysis (for more information please see the Supporting Material). There was also a large and significant positive correlation between performance on the RMIE task and GIDYQ score (i.e., the poorer the RMIE performance, the more the current gender dysphoric traits).

**Fig. 1:**
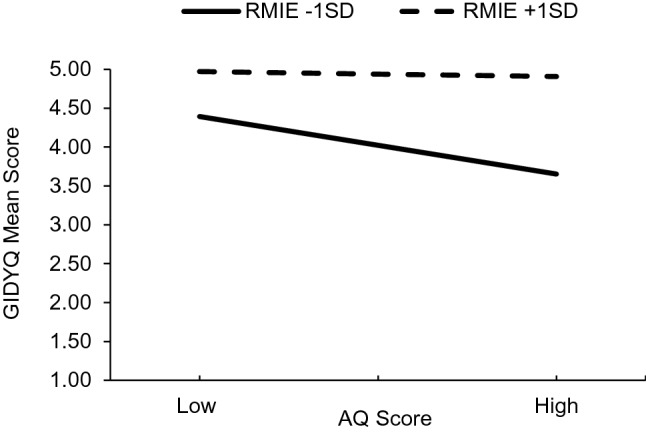
Regression plot showing the interaction between autism traits (AQ) and high/low performance on RMIE on current gender dysphoric traits (GIDYQ)

Next, we examined whether the association between AQ total score and GIDYQ mean score was moderated by performance on the RMIE task. The overall model of AQ score, RMIE score, and their interaction was significant, *F*(3, 97) = 42.51, *p* < .001, *R*^2^ = .57, and the interaction added *R*^2^ = .04 above the main effects. AQ score negatively predicted GIDYQ, *b* = − .03, *t*(97) = − 3.72, *p* < .001 and performance on RMIE positively predicted GIDYQ, *b* = .06, *t*(97) = 8.78, *p* < .001. As expected, the interaction between AQ and RMIE also predicted GIDYQ, *b* = .003, *t*(97) = 2.94, *p* = .004, indicating that mentalising moderated significantly the relation between autism traits and current gender dysphoric traits. Figure 1 depicts the effect of interaction on GIDYQ.

Simple slopes analysis showed that when performance on RMIE was low (−1SD), AQ score predicted negatively and significantly current gender dysphoric traits, *b* = − .05, *t*(97) = − 4.02, *p* < .001, whereas, when it was high (+1SD), AQ score did not predict current gender dysphoric traits significantly, *b* = − .004, *t*(97) = − .49, *p* = .625.

### Exploratory Analyses

Table 2 presents mean (*SD*) scores on the AQ, RCGI, GIDYQ, and RMIE among individuals who reported a formal diagnosis of autism and those who did not.

**Table 2:** Means (SDs) and inferential statistics for group differences

	Diagnostic group		Group differences		
	Autistic	Non-autistic	*t*	*p*	*d*
	(*n* = 13)	(*n* = 88)			
AQ	24.92 (5.56)	18.88 (7.24)	2.88	.005	0.94
GIDYQ	3.27 (0.81)	4.62 (0.55)	− 7.72	< .001	1.95
RCGI^a^	3.05 (0.37)	3.77 (0.60)	− 5.97	< .001	1.45
RMIE	14.15 (7.07)	25.50 (6.52)	− 5.79	< .001	1.67

*AQ* autism-spectrum quotient, *GIDYQ* Gender Identity/Gender Dysphoria Questionnaire, *RCGI* the Recalled Childhood Gender Identity/Gender Role Questionnaire, *RMIE* reading the mind in the eyes task

^a^Welch’s *t* test

In line with the findings from multiple previous studies, relative to comparison participants, autistic adults showed significantly higher AQ scores (in keeping with their diagnosis) and significantly lower RMIE scores (suggesting a mentalising difficulty). More important for the current investigation, participants with autism showed significantly increased childhood crossgender identity/behaviour and current gender dysphoric traits than comparison participants.

Across the total sample (*N* = 101), there were nine participants who scored less than or equal to three on the GIDYQ, denoting clinically significant levels of gender dysphoria. Seven (77.78%) of these nine participants reported being in receipt of a formal diagnosis of autism (see Table 3).

**Table 3:** Contingency table of participants who scored either below or above the threshold on GIDYQ by group

Group	Gender dysphoric traits	
	Above threshold	Below threshold
Autistic	6 (6.52%)	7 (77.78%)
Non-autistic	86 (93.48%)	2 (22.22%)
Total	92 (100%)	9 (100%)

*N* = 101

*Above threshold* more than three, *Below threshold* less than or equal to three

A Fisher’s exact test indicated that there was a significant association between the diagnostic group (autistic/non-autistic) and GIDYQ score (above clinical threshold/below clinical threshold), *p* < .001, phi = – .61. The odds of denoting clinically significant levels of gender dysphoria were 50.17 times (95% CI 8.49 to 296.31) greater for autistic participants compared to non-autistic participants.

## Discussion

In keeping with our predictions, this study found that AQ score was associated significantly with both the RCGI and the GIDYQ score, indicating that the higher an individual’s autism traits, the more their recalled crossgender identity and behaviour in childhood *and* the more their current gender dysphoric traits tended to be. To our knowledge, this is the first study to replicate George and Stokes (2018) findings of a significant relation between autism traits and current gender dysphoric traits in adults from the general population. It is also, the first study to demonstrate a link between autism traits and *recalled* crossgender identity and behaviour. This highlights a developmental continuity of the association, which is present in both children (Nabbijohn et al. 2018) and adults (current study and George and Stokes 2018). We also found that RMIE task performance was associated significantly with both the RCGI and the GIDYQ score (poorer mentalising = more recalled childhood crossgender identity/behaviour and more current gender dysphoric traits). Furthermore, RMIE task performance moderated significantly the relation between autism traits and current gender dysphoric traits. Specifically, only when mentalising ability was relatively low was there a significant association.

The results from exploratory group contrasts complemented results from the main association/moderation analyses; participants who identified as having a diagnosis of autism showed significantly diminished performance on the RMIE task and (clinically and statistically) significantly elevated levels of gender dysphoric traits. Of course, the results from these exploratory analyses should be treated with caution, given the small number of autism cases involved and the fact that participants were recruited via opportunity sampling, which may have biased selection (i.e., perhaps those individuals with autism who also had gender identity difficulties were attracted to take part in the study, given its focus).

From a theoretical perspective, the results of the current study come to add to the discussion on whether the link between autism and gender dysphoria exists purely at a behavioural level (Turban 2018; Turban and van Schalkwyk 2018). Our findings indicate that mentalising *moderates* rather than explains the relation between autism traits and gender dysphoric features. As such, it could be argued that, in autism, a weakness in the process of mentalising may contribute to increased fluidity of gender identity. A child in whom the process of mentalising is diminished/atypical is likely to experience elevated feelings of gender fluidity as they fail to internalise a typical proportion of the attributes that stereotypically define their biological sex. It is also possible that this child in whom the experience of guilt and embarrassment is reduced will be less affected by societal pressures and prejudices and therefore less likely to desist crossgender behaviour as they develop crossgender interests.

Nonetheless, it is unclear whether this approach accounts for the conviction and conduct of those who assume gender roles out of keeping with their biological sex (i.e., trans-gender individuals/individuals diagnosed with gender dysphoria). Could it be the case that increased autism traits in people with gender dysphoria reflect genuine autistic features *only* when mentalising difficulties are present? This question remains open until directly investigated.

One thing to note here is that that participant recruitment was conducted using an online platform. Although online studies yield results that are equivalent in terms of their reliability with other widely employed methods of data collection (e.g., Buhrmester et al. 2011), firm conclusions might await independent replication of the current findings in studies that employ multiple methodological approaches and sampling procedures. Moreover, future research might usefully examine the extent to which autism and gender dysphoria share underlying causes at more than one levels of explanation (biological, genetic, environmental), in order to provide insight on the extent to which there is true comorbidity between them (Williams and Lind 2013).

In conclusion, the current study shows for the first time (a) an association between autism traits and recalled childhood crossgender identity, (b) an association between gender dysphoric traits and mentalising, and (c) that the link between autism and current gender dysphoric traits is moderated by mentalising skills in the general population.

## Electronic supplementary material

Below is the link to the electronic supplementary material.

Supplementary file1 (DOCX 24 kb)
