# Supplementary material for: Mentalising Moderates the Link between Autism Traits and Current Gender Dysphoric Features in Primarily Non-autistic, Cisgender Individuals
Source: J Autism Dev Disord. 2020 Apr 1;50(11):4148–57. doi: 10.1007/s10803-020-04478-4 (PMC7560908; doi:10.1007/s10803-020-04478-4)
Supplement: Supplementary file 1 — Supplementary file1 (DOCX 24 kb) [file 10803_2020_4478_MOESM1_ESM.docx]

**Supplementary material**

1. **Statistical analyses excluding participants with autism**

When participants who reported possession of a formal diagnosis of autism (*n* = 13) were excluded from the analysis, results did not change substantively except in one analysis. Just as in the full sample, AQ remained correlated negatively and significantly with both GIDYQ, *r* = -.26, *p* = .017 and RCGI, *r* = -.30, *p* = .005, after the exclusion of these 13 participants. Moreover, RMIE remained positively and strongly associated with GIDYQ, *r* = .59, *p* <.001. The interaction between AQ and RMIE predicted significantly GIDYQ, *b* = .003, *t*(84) = 2.38, *p* = .019. Simple slopes analysis showed that when performance on RMIE was low (-1SD), AQ score predicted negatively and significantly GIDYQ, *b* = -0.04, *t*(84) = -3.30, *p* = .001, whereas, when it was high (+1SD), AQ score did not predict GIDYQ, *b* = -0.003, *t*(84) = -0.31, *p* = .761.

The *only* result that changed substantively (i.e., from significant to non-significant, or vice versa) in this reduced sample of 88 participants was the RMIE × RCGI correlation. In the full sample, the correlation was moderate and statistically significant, whereas in the reduced sample it was small and non-significant, *r* = .14, *p* = .201. A power analysis using G*Power 3.1.9.2 indicated that to detect an association of .33 between RMIE and RGIQ on 80% of occasions (as recommended by Cohen, 1992) using two-tailed tests, 69 participants were required. Arguably, it seems likely that the correlation we found between RMIE and RGIQ in the full sample was inflated by the inclusion of autistic participants.

1. **Correlation analyses among AQ subscales, GIDYQ, and RCGI**

An anonymous reviewer made a constructive suggestion that we conduct additional analyses to explore the relation between gender dysphoric traits, on the one hand, and each of the individual AQ subscales, on the other hand. We have done this and reported the results in supplementary Table 1, below. However, we think it is important to make clear that these analyses are entirely post hoc and we did not have any predictions about the results. Exploratory analyses that are not based on a specific prediction (following from a sound theory) risk contributing to an inflation bias (i.e., selective reporting/p-hacking; e.g., John, Loewenstein and Prelec 2012; Kühberger et al. 2014; Masicampo and Lalande 2012) in the field. If researchers regularly analyse variables they never intended to analyse it will inevitably lead to the publication of false positives. In turn, if other researchers then make predictions based on results that are (conceptually and statistically) likely to reflect type II errors, then the field becomes (further) biased toward the search for and belief in positive results. Therefore, we urge readers to be cautious when interpreting the results of these supplementary analyses. That being said, George and Stokes (2018) did analyse the relation between GIDYQ score and each subscale of the AQ (although it is not clear the analyses were based on specific hypotheses) and found that all of them were significantly associated with gender dysphoric features. The current results replicate the significant association between AQ communication subscale and GIDYQ. This suggests that there *may* be a reliable connection between gender dysphoric features and communication ASD features, specifically.

| Supplementary Table 1 | | | | |  |  |  |
| --- | --- | --- | --- | --- | --- | --- | --- |
| *Bivariate Correlations among AQ subscales, GIDYQ, and RCGI* | | | | | | | |
| Variables | 1 | 2 | 3 | 4 | 5 | 6 | 7 |
| 1. AQ social skills | - |  |  |  |  |  |  |
| 2. AQ attention switching | .45*** | - |  |  |  |  |  |
| 3. AQ attention to detail | .05 | .16 | - |  |  |  |  |
| 4. AQ communication | .64*** | .44*** | .21* | - |  |  |  |
| 5. AQ imagination | .29** | .09 | -.05 | .39*** | - |  |  |
| 6. GIDYQ | -.04 | -.10 | -.12 | -.50*** | -.31** | - |  |
| 7. RCGI | -.28** | -.29** | -.08 | -.35** | -.04 | .53*** | - |
| *Note.* *N* = 101; AQ = Autism-spectrum Quotient; GIDYQ = Gender Identity/Gender Dysphoria Questionnaire (low scores = more gender dysphoric traits); RCGI = The Recalled Childhood Gender Identity/Gender Role Questionnaire (low scores = more childhood crossgender identity). | | | | | | | |
| **p* < .05,***p* < .01, ****p* < .001 | | | | | | | |
